# American Medical Association, Section on Stomatology

**Published:** 1903-09

**Authors:** 


### Reports of Society Meetings.

### AMERICAN MEDICAL ASSOCIATION, SECTION ON STOMATOLOGY.

THE meetings of the Section on Stomatology were held in the Touro Synagogue, Carondelet Street, near St. Joseph Street, New Orleans, May 5 to 8, 1903.

### FIRST SESSION.

The meeting was called to order at 2.25 P.M. by the Chairman, Dr. M. L. Rhein, New York. The secretary, Dr. Eugene S. Talbot of Chicago, reported as follows:

The programme of the Section is published in the general programme of the American Medical Association. We are unfortunate in some respects in regard to this programme. I had spent a great deal of time in trying to get out one which would be superior to anything heretofore produced in this direction, and I think we have an excellent one. Each of the authors invited by me to present papers was asked to bring out a certain line of thought; and, unfortunately, some of these people, from one cause or another are unable to be with us,—in two cases on account of sickness, in two cases on account of legislation concerning dental laws, and some have gone to the International Medical Congress in Madrid. I have nearly all the papers with me, and I would make a motion that this programme as it is be adopted as a whole. Seconded and carried.

I will make another motion, that as many of the papers be read as possible of those who are not present. Seconded and carried.

I would also move that Dr. W. E. Walker, of New Orleans, be placed upon the Executive Committee to fill the place of Dr. Andrews, of Cambridge. Seconded and carried. An effort was made to have Dr. Gilmer accept membership in the Executive Committee, but he asked that his name be withdrawn. Dr. M. H. Fletcher, of Cincinnati, was then reappointed.

The Chairman, Dr. M. L. Rhein, of New York, then read his Address.

(For President Rhein's Address, see page 449.)

DISCUSSION.

*Dr. M. H. Fletcher,* Cincinnati.—The subject presented to us lies near to my heart in the practice of our specialty. I fully agree with the ideas advanced; at the same time it seems to me there is a channel in which we should work in order to bring the matter before the public as it should be. We all know how long it takes the general public to receive a new fact of any kind. Everybody used to believe the world was flat. Darwin's theories have only begun to be accepted by the best people. I believe that if it were possible for us to so advertise the simplicity of this thing with the general public the matter would advance in favor much more rapidly. All dentists know that such a thing should be done. To properly introduce such a plan we would have to begin with our own students. Those of us who are teachers should impress it more fully upon students at the beginning. We should teach the general public that the simple fact of cleanliness prevents disease more than anything else. Until we can impress this fact I do not think we are apt to make very rapid progress. The insertion of fillings and the treatment of diseases of the mouth seem to me of minor importance compared with this. I think it is becoming of us as a company of progressive men to give our endeavors to the accomplishment of some plan whereby the suggestions of the Chairman's Address can be carried out not only by the dentists, but by the general public. I want to say again that I approve of the paper in its entirety.

*Dr. Fredericks,* New Orleans.—Prophylaxis in the case of the teeth, and in everything else respecting human nature, seems a good thing and we ought to endorse it. These things ought to be done, but we do not do them. For instance, we will sometimes see an educated physician, one who knows the consequences of the morphine or alcohol habit, make use of them and become their slave. On the other hand, to show what nature does for us, look at the negro's teeth, which oftentimes never have a tooth-brush on them. If we followed nature more closely there would be less need for prophylaxis.

*Dr. M. L. Rhein.*—In setting forth this proposition it has been my object not to enter into any question of etiology, or methods of treatment, or anything of that kind, but to consider simply the advisability of adding to the dental laws in the various States a clause, not necessarily permitting the employment of dental nurses, but defining their limitations, making it apparent that they are legally entitled to be of use. I am certain that as a physician, if I desire to employ a nurse for this purpose, I have the same right to that use as I have in any other capacity as a physician, and I am sure I could carry my point legally; but it is my desire to have their sphere well defined, so that there would be no obection to their use by the general dentist. I do not care to enter into the question of how difficult it is to bring a new plan before the public, but in my own mind I am certain that if my plan meet with general approval, the next step will be easily accomplished,—that is, the addition of a training-school for dental nurses to the regular training-school for nurses. I trust that the gentlemen, in order to facilitate the other business, will give the Section the benefit of their thought on simply this particular point.

*Dr. George F. Eames,* Boston.—As is the case to-day and at other times, whenever Dr. Rhein gives us a paper he covers the ground so thoroughly, and his views are so in accord with my own, that I am unable to say anything except a word of commendation. I have in my practice carried out many of the principles which he has enunciated here, and have trained assistants to supplement me, rather than to take entire charge of what we may call prophylaxis. When I begin a prophylactic treatment of the mouth my assistant usually sees what I do. I take opportunity then to explain what is to be finished, or I direct such an assistant to do such and such things. The matter is perhaps not exactly to the point, and yet to the point of the desirability of having regularly appointed dental nurses; that is, the education of the public as to the desirability of this, it seems to me, comes under the head of a principle which all stomatologists should endorse and have carried out from the beginning of their practice. There is no section of work that can require more blind confidence and trust in the practitioner than dentistry. I often say to my patients that unless I can be the doctor and they trust me as such, believing what I do is for their best good, I would rather give up my profession. When I assure them that in the prophylactic treatment of the mouth I am doing the very best I can possibly do for any patient, and the most important work that can be done in the mouth, they must believe me and co-operate with me in that work. That done, the rest becomes easy. When a graduate receives his diploma from a dental college, or from any institution, it grants him certain privileges, but it is understood that with these privileges within the law there are also certain restrictions. It is so with the appointment of graduate nurses or dental nurses. They do have the privilege of doing certain things within the mouth under the direction of the stomatologist, but there are restrictions to this as well as to other privileges. It seems to me we cannot but endorse the plan that has been suggested by our chairman.

*Dr. Gilmer,* Chicago.—I am thoroughly in accord that it is important, that it is necessary, to have a better way of accomplishing the work spoken of than by the methods that now prevail. How we can bring that about I do not know. It would be better, it seems to me, to have specialists for that work. The treatment of diseases of the mouth offers a very large field. It does not seem to me that we could trust the treatment of the so-called pyorrhœa alveolaris, except the merest first cleansing or some subsequent medication, to any one who is not thoroughly skilled in that work. I consider this treatment one of the most difficult things to perform,—that is, the thorough removal and the surgical treatment of this so-called pyorrhœa alveolaris.

As to the training of nurses in the hospital for certain work, with this I am in accord; and my connection with hospital work and the teaching of nurses give me some positive ideas on that subject. I very thoroughly approve of the training of nurses in the care of the mouth, in the preparation of the mouth previous to surgical operations, and in the various diseases, and I think we should continue the education of the people by personal advice. I believe I save more teeth and do far more good in the prevention of disease of the mouth by constant demonstration and insistence on the care of the mouth by the patient than by any other means.

*Dr. Fredericks,* New Orleans.—I do not find fault. "Cleanliness is next to godliness," and if it could be carried out it would be conducive to health. The chairman's paper deserves all the commendation and praise; at the same time, we must look at the other side.

*Mr. T. E. Constant,* Scarborough, England.—Mr. President and gentlemen, I do not know that I can add anything to what has been said by the previous speakers, because I think that no dental surgeon would hesitate for a moment to endorse everything that our president has said in connection with the matter. None of us have any doubt at all that the public would be greatly benefited by such a scheme. But, of course, our laws in England are such that the education of such a body of semitrained people might act injuriously. Whether this would be so in this country I am not in a position to say; but that is the aspect of the question that we would have to consider. On the broad question as to whether the suggestion is a good one for the public generally, I think there could be but one answer,—that which has been given by the other speakers.

*Dr. W. E. Walker,* New Orleans.—Mr. Chairman, I thank you for the privilege of speaking simply a few words of commendation. I do not see any harm that could come from the plan, and I can see infinite good to come from it. When in general practice a very large portion of my time was spent in doing the very work that the paper suggests, having great assistance in the work by lady helpers. While I would certainly agree with Dr. Gilmer that the treatment of pyorrhœa alveolaris from the surgical standpoint would be rather unwisely placed in the hands of such semitrained assistants, at the same time they could be of great assistance if used somewhat as Dr. Eames says, to supplement the work of the dentist; and the paper does not carry with it anything more than a proposition to supplement, with the addition of legalizing, their practice. As the doctor said, he has a right to employ trained nurses to help in this work, whether they come from a training-school or have been trained individually. But the idea seems to make it beyond the possibility of criticism on the part of the dentist who is not a physician, that he may freely employ these trained assistants in his office and not run any risk of criticism or legal complication. It would certainly be much better than employing undergraduates that you cannot hope to keep with you more than a year. I certainly cannot see any objection to it, and I hope the Section will endorse the idea.

*Dr. H. E. Belden,* New Orleans.—The ideas are in line with my views, and I think we should have dental assistants to carry out our wishes in cleaning the teeth. The cleansing is an irksome task on us and demands a great deal of attention. The lines for a livelihood are closely drawn, and we have to devote ourselves to the wider tasks in our profession, and if we can put off these duties which can be performed by assistants, I think it becomes us to do so. We help our patients by giving them treatment by our assistants carried out under our supervision. The paper is in accord with my views on the subject.

*Dr. C. V. Vignes,* New Orleans.—I think the idea of having such trained assistants is a very good one. From a legal standpoint, I do not know what condition exists in England, but I know that here any body can practise that which he may deem essential. It is suggested that these special nurses be trained, because the young man or graduate at the end of a few months would go into general practice. I believe that the trained nurse would do about the same thing. She would go into general housekeeping. I do not know how it obtains in other parts of the country, but down here trained nurses are pretty liable to get married, and the vocation is a stepping-stone to matrimony. I believe the field is a large one for young men, and that it would assist a great many to earn an honest living and keep them in the straight path; that is, they would practise in an ethical manner if they had the right start. I think it is an opening, not so much for the trained nurse as for the benefit of the young dentist who has a very hard road to travel.

*Dr. Eugene S. Talbot.*—I have no fear as to the future education of the dentist. The experimental stage is passed. Dental education commenced to shape itself when universities established dental departments. Although for fifteen or twenty years after that time there seemed to be a chaotic condition, yet the past five years has worked a wonderful change in the department of dentistry. The adoption of a four years' course is the culmination of the educational problem from a dental stand-point. The university will place this department of medical science on an equal footing with other specialties of medicine. The Section on Stomatology of this Association has played a prominent part in shaping dental education in America, and I for one am perfectly willing to let the universities work out the problem.

Credit should be given the late Dr. A. C. Hart, of San Francisco, for suggesting the cleansing and polishing of the surfaces of the crowns of teeth. Scientifically he did more in the study of this method than any individual. His death was a result of his enthusiasm along this line of investigation.

I agree with Dr. Rhein in relation to specially prepared women for the care and cleansing of the teeth as first suggested by Dr. Wright, of Cincinnati. That such women should be connected with hospitals to care for the mouths of the sick is certainly very important. How physicians can expect to successfully treat their patients with uncleanly mouths is a problem. It would seem to me that this paper should not only be published in the *Journal of the American Medical Association,* but later in all medical journals in this country and in Europe, and that reprints should be sent to all the hospitals. Every physician should read it. The dentist should insist on his patients coming to his office every three months to have the teeth cleansed. When the patients understand that by so doing they are saving dollars in fillings, etc., they will readily acquire this habit. If all dentists would do this, the public would be educated soon to this method.

*Dr. Rhein* (closing).—I desire to take up just a few moments of your valuable time in closing the discussion of my address. I have tried not to take up the time of the Section with any exhaustive treatment of the subject, and it has been my desire not to go into the various influences that would be felt from the pursuance of such a plan as I have formulated. In my practice the plan of the return of the patient, as mentioned by Dr. Talbot, is pretty well in effect. Every one of my patients is sent for at regular intervals. The presence of caries is not considered. The removal of the racemose organism, which is so painful in its effects in early life, and of the plaque formations are equally important.

In regard to Dr. Gilmer's remarks, I do wish to say that it is far from my purpose to permit a trained dental nurse (if I may use the term) to perform what he and I believe all dentists consider one of the most serious and difficult surgical operations. That such people should not be permitted to work in that condition or in the ordinary forms of dental work is the main object I had in introducing this matter and in asking for your approval of the suggestion to legalize the position of a dental nurse so as to legally establish the limts of her sphere of usefulness. Personally I would prefer that these assistants be women, the reason for which I will not take the time to explain. In my opinion, they might be permitted to remove deposits in normal mouths, but in distinctively pyorrhœal cases their duty would come only after the surgical work had been completed by the surgeon, in following his direction in thoroughly polishing the tooth-structures and in carrying out any medication under his direction. I desire to strongly emphasize this. In conjunction with this comes up the point raised by Mr. Constant. His objection to its usefulness is based upon the condition of legislation which does not exist in the United States. I understand that in England the passage of such a law might give an improperly trained person an interest in dental practice and an improper opportunity to work their way before the public without being interfered with by the law. In the United States, in most of our States, at the present day the laws are rigidly enforced. This is especially true in the eastern part of the United States, and here it would be impossible for infractions of the dental law to be performed. I would also say, in reply to Dr. Gilmer, that it is a great pleasure to me to know that there is one hospital in the United States where the mouth is properly prepared preliminary to the surgical operation. I know of no such condition in the city of New York. In that respect we are rather behind the West. Outside of that one hospital, I think the general surgeons in Chicago or of the Northern cities do exactly what the general surgeons do in the East: when they open the peritoneum, when they have an operation for appendicitis, or a laparotomy, the condition of the patient's mouth is not considered. That has been my experience in the East, and I have seen some such conditions in the West. I do not for an instant suppose that any of us who do surgical work would permit any patient of ours to go on the operating-table in such condition. I refer to those medical men who are absolutely devoid of the ordinary rudiments of knowledge as to the oral conditions and the septic influences that may proceed from the oral cavity. I thank the members heartily for the kind approval which they have given the address.

Dr. Talbot moved that a committee of three be appointed to consider the suggestions of the Chairman's Address and to form resolutions. Seconded and carried. Dr. George F. Eames, of Boston, Dr. W. E. Walker, of New Orleans, and Dr. Eugene S. Talbot were appointed such committee.

Dr. Talbot offered a resolution that Mr. T. E. Constant, of Scarborough, England; Dr. A. C. Spaulding, of Paris; Dr. D. E. Causch, of Brighton, England; Dr. Michael Morganstern, of Strassburg, Germany; Dr. Oskär Römer, of Strassburg, Germany; Dr. H. Pichler, of Vienna, be made members, by invitation, of the Section on Stomatology. Seconded and carried.

It was moved and seconded that the following gentlemen be recommended as associate members of the Section on Stomatology: Dr. H. E. Belden, Dr. C. Edmund Kells, Jr., Dr. Paul de Verges, and Dr. Jules J. Sarrazin, all of New Orleans. Seconded and carried.

### SECOND SESSION.

The second session of the Section on Stomatology was called to order on Wednesday, May 6, at 2 P.M., the Secretary, Dr. Eugene S. Talbot, in the Chair.

A paper on the "Tolerance of the Tissues to Foreign Bodies, with Special Reference to the Pulp and Gums," was read by Dr. M. H. Fletcher, of Cincinnati.

(For Dr. Fletcher's paper, see page 596.)

### DISCUSSION.

*Dr. Eugene S. Talbot.*—In the discussion of Dr. Fletcher's paper I am obliged to take issue with him in a number of points. While it is possible that gutta-percha will be tolerated at the apical foramina more readily than a steel broach, yet to understand the pathology arising from such irritant we must first take into consideration the structures involved. We cannot compare the peridental membrane and the alveolar process with other structures of the body. The alveolar process is a transitory structure; the tooth to a certain extent is a foreign body. The alveolar process and peridental membrane, unlike the other structures, has not come to stay. It is waiting for the least irritation to set up absorption and destruction of the peridental membrane. While it may tolerate gutta-percha better than metal, in either case the irritation produces absorption and destruction. It will not encapsulate foreign substances like other tissues. I have demonstrated this many times in many ways. Neither can we contrast a pulp like that of a human tooth with that of an elephant's tusk or a pulp that is persistent, as I showed in my paper last year. The pulp is practically a degeneration, even in health. The expansion and contraction is nil, hence pulp-stones and frequent inflammation resulting in abscess of the pulp without pain. The restricted portion of the apical end of the root restricts repair. The constitutional causes of interstitial gingivitis have local action because the gums are a secretory structure, and because of the transitory nature of the gums and alveolar process, next to the nervous system, they are always the first involved. The arteries, nerves, and veins terminate in this structure. They come up against a blank wall or nearly foreign substance (the root of the tooth), hence it may be called a terminal structure. I agree with the essayist that tartar invariably produces interstitial gingivitis, and in that case is entirely local in its origin, but the constitutional variety may best be illustrated in something like eighteen or twenty syphilitics that I have had in my practice for a number of years. By local treatment I am able to practically restore the gums and alveolar process to health, and the patient leaves my office after a given length of treatment feeling finely, but they invariably return, sometimes in six months, a year, or two years, with marked interstitial gingivitis. No matter how well the patient may be in a general way, here we have marked illustrations of the constitutional variety. What is true of syphilitics is also true in consumptives, diabetics, and auto-intoxications of all varieties. The calcareous deposits are no doubt irritating to the surrounding tissues. They should in all cases be removed. I wish to congratulate Dr. Fletcher on the admirable manner in which he has handled the subject.

*Dr. A. E. Baldwin.*—I am not sufficiently conversant with many of the points made by the essayist and Dr. Talbot to discuss them in their entirety and would want to thoroughly digest the material presented before I would feel competent to discuss it intelligently. There are one or two things in the discussion by Dr. Talbot that strike me from a little different stand-point, and this brings me to the point spoken of in our Saratoga meeting. I think we as a profession, belonging to the great medical ranks, ought to define our premises a little more closely than we do, and then we could have much more intelligent and accurate discussion. As, for instance, we speak of degeneracy being a change from the normal. The first essential is to fix upon the data for the normal. Without this we would be discussing largely in the dark, because every one's stand-point would be different as to the point taken for normal. The statement has been made that the pulp itself is a degenerate condition. Before I could accept any such statement I should want to know very definitely what is considered normal.

The essayist speaks of the tolerance of the tissues for certain things, and mentions an illustration of seventeen years' standing. I think the position of the essayist that there is an apparent tolerance is well taken. I had a similar case in my own experience in a left lateral superior incisor. After two or three years I removed it because I was conscious, in examining the mouth, of a little projection under the lip. There was no irritation. I removed the substance and intended to make an examination of it, but in the moving of my office it was lost.

I would be very glad to hear from Mr. Constant, our visitor from abroad.

*Dr. Curtis.*—The tolerance of the tissues for foreign substances varies, I believe, according to the nature of the substance. The doctor has brought that out very clearly and demonstrated that the bullet or similarly irritating substance can be tolerated for a long time. I have in mind a case brought to my attention of a child who stepped upon a headless pin, and by the time a physician reached the house it could not be found. Three physicians consulted upon the advisability of operation, and three months later the pin came out at the hip. The patient found that the clothes were cut. There was slight inflammation, and the pin was pulled out by the trephine, and no trouble at all resulted from the accident. I know a gentleman who has a very large hypodermic needle, an aspirating needle, in his leg, and it has been there for some twenty years. Evidently it has lodged against the bone and does not take the usual course of passing through the tissues. I suggested the use of the X-rays, but he said he did not want them, and that he would be more comfortable if it were not located, as he was in good health.

I cannot agree with the point taken by Dr. Talbot as to the tooth being a foreign substance, unless, perhaps, he considers the tooth a degenerated foreign substance. I believe that when the tooth begins to develop it begins to degenerate.

I think gutta-percha is better tolerated than any other substance. Some instructive lessons might be gained from the use of the X-ray.

The syphilitic phase of interstitial gingivitis is one that for many years has interested me. I have felt that many if not all the recurrent cases of gingivitis are due to syphilis. When the inflammatory process returns after carefully administering proper treatment, I have found that the cases usually do well under syphilitic treatment, and in a great many such cases have proved beyond doubt the presence of syphilis. It has not always been acquired by the patient, but was sometimes inherited. We know that the child of a syphilitic parent, either father or mother, has syphilis. It is a positive fact that the children inherit the diseases of the parent which are present at the time of conception, and that the mother usually becomes diseased at the time of gestation. These cases are perhaps the most difficult to treat because of the lack of definite knowledge. I have gained my knowledge more particularly through the study of life. The germ or spore of syphilis is detected in the blood. A Russian explained this in his text-book written long ago, but being before the time of the microscope, he was unable to give us definite data by which we could conduct our experiments. I believe that wherever I find the syphilitic spore the patient bears the mercurial treatment well, and if placed upon very mild preparations of small doses of the red oxide or biniodide of mercury they do well. I usually give a sixteenth of a grain three times a day, increasing it two or three times. If the treatment is persisted in for months, you can eliminate the degenerative condition which maintains this syphilitic gingivitis.

*Dr. M. L. Rhein.*—I want to compliment Dr. Fletcher upon the admirable paper presented. I regret that I find myself somewhat in the same position with Dr. Baldwin in being unable to discuss the paper as fully as I would like. I was especially pleased with a few little technical points in the essay. We have at times been accustomed to the use of terms that were rather exaggerated, and I was very much pleased to hear Dr. Fletcher make use of the term "sapræmia," instead of septicæmia. I am convinced that we get very few cases of real septicæmia such as the one he cited, which is a distinctive exception to the general run of cases which are sapræmic in their nature. We are here for discussion, and from a surgical stand-point I take exception to the view of the essayist regarding the after-effects of extraction of a tooth under the circumstances cited. I cannot conceive of a case of this nature in which surgical interference can be objectionable or be a potent cause for evil. I was considerably astonished to see the essayist take the view which he did,—that the extraction of this tooth may have had or did exercise a considerable bearing on the fatal ending of the case. It seems to me contrary to all the accepted surgical principles at the present time. Furthermore, it brings up the question frequently met with, whether because of the presence of pus it is not advisable to delay the extraction of a tooth, supposing it to be determined upon. This is a question well worth the consideration of this Section.

I agree with the essayist, and am opposed to the view of Dr. Talbot of the tolerance of foreign materials in the vicinity of the peridental tissues. My own clinical experience with the use of gutta-percha and my examination and careful observation of hundreds of cases leads me to coincide very thoroughly with Dr. Fletcher that gutta-percha is tolerated in the most remarkable manner by the soft tissues. I could cite a number of interesting cases bearing out this view. If there is a substance which the tissues will tolerate more freely than gutta-percha, it is perfectly fused porcelain. I have demonstrated that by the magnificent tolerance shown by the tissues in the amputation of a necrosed root in one of the multirooted teeth, attaching a porcelain root to such a crown. The observation of these cases for three or four years shows such a remarkably normal type of healthy gum tissue around the porcelain root as to thoroughly endorse the general proposition laid down by the speaker.

I rather regret that Dr. Curtis brought my name into his view of the syphilitic origin of so many cases of interstitial gingivitis. While I am thoroughly aware that syphilis plays an important rôle as a cause of peridental pathological conditions, I am totally opposed to the extreme view that Dr. Curtis takes on this question, in which he says that almost all cases of recurrent pyorrhœa or interstitial gingivitis will show a distinct syphilitic origin. I also do not coincide with his views that the syphilitic germ can be for a certain number of years demonstrated in the blood. Dr. Lisquaid claimed to have made this discovery twenty-five years ago, but it is well known that the matter is disputed at the present day. The point hinges upon our ability of diagnosing a pre-existing syphilitic state by a blood examination, and I do not care in any implied way to appear as accepting the view laid down by Dr. Curtis.

*Dr. Hans Pichler,* Vienna.—Besides gutta-percha, paraffin is well tolerated, as the large surgical uses of this substance show. Dr. Trauner, of my city, has used it for the filling of old cavities with open foramen before the development of the teeth is completed, and he has had good success.

*Dr. Curtis,* New York.—I think the question of extracting the teeth in the presence of inflammatory processes would depend entirely upon the individual case. You can lay down no rule whatever for such extraction. In this case I have no doubt the extraction did much to hasten the fatal ending. I think there evidently was an acute inflammation, or acute infection, lighted up by reason of opening new tissue to this disease, against which the physician and patient were battling. I therefore believe it better to temporize with these cases and try to tolerate this inflammatory process until the greater part of the other trouble has been disposed of. Ordinarily speaking, I think the extraction of these teeth is wise, but never when the extraction is liable to extend the inflammation.

*Dr. George F. Eames.*—It seems to me that the opposite views expressed on this paper have a great deal of truth in them, because we can all in our experience recall cases of great tolerance of the substances named by the essayist. We can also recall instances in which there has been little tolerance to foreign bodies, and when Dr. Talbot said that when we consider the tolerance of foreign bodies in these tissues we must take into consideration the structures involved, he would have done well if he had said, we must take also into consideration the general condition of the individual. It seems to me that explains the very great difference of patients in tolerating not only foreign bodies, but all toxic irritating substances. The case of death to which reference has been made I think should not be classed as rare. I believe there are a great many cases of death from autointoxication from this condition of inflammation in which pus is swallowed and fresh infection caused, and in which the diagnosis has been entirely different. I remember a case in which a patient suffering from gingivitis made the remark that she was in no fit condition to go to a dentist. My answer was that if she did not see fit to come to a dentist, she would have to have the undertaker. The attending physician did not realize that the condition of the mouth was bringing death to the patient.

As to the extraction of teeth in the condition named by the essayist, while it would seem that the surgical principle of removing the irritant at once should obtain in most dental cases, yet we have on record a number of cases in which very serious results have followed the extraction of teeth. We have to recognize that when another tooth has been extracted the poison has continued and great destruction followed. I believe this must be due to fresh infection of a surface reopened, and that there is some truth in the statement that we should get rid of the infection by some other means.

*Dr. M. L. Rhein.*—I take the liberty of speaking again in regard to this method of extraction. I certainly feel from considerable observation that all such cases as stated by Dr. Eames and Dr. Curtis are not due to the question of extraction, but due to the faulty surgical treatment involved in such cases.

*Dr. W. E. Walker,* New Orleans.—I have always felt, in cases in which death was stated to be due to extraction, that the position just taken by the Chairman is a correct one,—that death is not due to extraction, but to the faulty technique. Who can say in many cases that death would not have resulted had extraction not been done. In many of these cases of osteomyelitis simply removing the tooth is not sufficient. The marrow of the bone is permeated and should be treated. The whole mouth should be treated wth antiseptics, and the socket as well. Considering the tooth as a foreign substance, as Dr. Talbot has said, and as being the cause of gingivitis, is, I think, placing the cart before the horse. I believe if we were to use the word inflammation more carefully, and not consider as inflammatory conditions those which are simply passive hydræmia, we would be more correctly understood. In cases of so-called gingivitis, or Riggs's disease, in the early stages, I believe the tooth primarily is not an irritant, is not a cause, and is not a semiforeign substance, but that the disease is due to some local condition, together with lack of exercise calling for proper nourishment. Again, there is some systemic condition. In the majority of cases we have an accumulation of the blood on the venous side of the circulation, and because there is not the normal stimulus due to mastication the faulty general circulation becomes more manifest in the gum tissue. There is a lack of the normal stimulation of the vasomotor system, and this passive hydræmia keeps the tissues overfilled with blood of a venous variety which does not contain oxygen, and the tissues, though abundantly supplied with blood, are starved. The condition may be likened to Canal Street, where there may be a great mass of people but less business being done than if there were but one-half the number on the street. We know that the cementum contains hundreds of layers which have been put on during the life of the individual. This I think seems to demonstrate the fact that the fibres of the cementum have but a limited life, that they cannot hold on indefinitely to that tooth and hold it in its socket, and that additions of fresh cementum entangle fresh fibres to hold the tooth. The old fibres lose their life and a separation occurs. The connective tissue is exposed, and we have weeping of the serum. The bone is not nourished, and this serum in oozing out contains more lime-salts than the serum would ordinarily contain, more than the blood would contain. There is the additional mechanical irritation of the calculus, and a separation of the tooth results.

*Dr. Eugene S. Talbot,* Chicago.—It is very necessary that I should go on record regarding this matter. Dr. Walker misunderstood me. I did not intend to say that the tooth as a foreign body produced irritation. It acts somewhat as a blank wall. Otherwise I agree entirely with what Dr. Walker has said.

*Dr. Fletcher* (closing).—Dr. Talbot's position as to the transitory condition of the alveolar process of the tooth seems to me to apply to the hair and nails, to the tubercle, the pulling of the muscles. I think the law is established that where you have the attachment of a muscle you have these tubercles formed. If the human nail is not transitory why is it not like the baboon's, twice as thick. The alveolar process and tooth must be looked upon as transitory structures, but I fail yet to see why they are not susceptible to repair as long as they are present. It is a fact that the organism of the universe is probably losing certain structures for the benefit of the organism itself. With all the doctor's work I am stubborn enough to say that I have failed to be convinced that the transitory structures need necessarily without local irritation be subject to disease more than other structures. I should hope to be convinced if I am wrong.

The matter of systemic production of interstitial gingivitis by medicines, by syphilis, or by other systemic causes seems to me so absolutely difficult to prove that I must take the opposite position until they are proved. I would like to see a series of experiments carried on bacteriologically with the infection of the healthy gum. We would then have a basis for an opinion. While I admit that the gentlemen have reasonable grounds for their opinions, I feel that I have reasonable grounds for the other side.

In regard to my position about the extraction of the tooth, my paper did not give sufficient details, for the gums and alveolar process were in the worst possible condition and the gums of all the teeth were sloughing. It is possible, as Dr. Walker suggests, that the patient may have died without the extraction. Knowing the patient as I did, I believe, had the treatment been temporized as suggested by Dr. Curtis, the life might have been spared, but my counsel in the case was not heeded. The older surgeon was treating the case for acute tonsillitis.

*Dr. Rhein.*—Do you suppose with the condition of the worst possible kind that after removal of the tooth, if the proper germicidal agents were kept in that socket for a sufficient length of time, there would be any possibility of infection in that particular case?

*Dr. Fletcher* (answering Dr. Rhein).—I believe so, because I believe it is practically impossible to sterilize an open bone unless you have removed it. The deeper you scrape, the deeper and larger the blood-vessels in that bone get to be, and I believe that the blood clot may become septic in the mouth. I may be wrong, but I do not see how we would be able to make such a condition absolutely aseptic. I will admit that if it could have been kept perfectly aseptic this may not have added to what I believe was the cause of death. I think the extraction of the tooth with the addition of the septic condition turned the case downward instead of allowing it to recover.

DISCUSSION OF MR. CONSTANT'S PAPER, "THE DENTAL PULP, VIEWED WITHOUT THE MICROSCOPE."

(For Mr. Constant's paper, see page 411.)

*Dr. Eugene S. Talbot,* New York.—Just where the dental papilla leaves off and the dental pulp begins is a question worthy of discussion by competent men. Why the formative should possess two distinct names I am unable to state. What is called the dental papillæ still requires a large amount of research work especially along the line of histology. I am glad that Mr. Constant has given so much thought to the dental pulp. We as practitioners of stomatology have not laid stress enough upon this organ from a scientific point of view. This I have stated before, and it was for the purpose of bringing this organ more forcibly to the notice of the profession that I had instituted these symposiums from year to year in our proceedings, and I trust that more work will be done upon histology, physiology, and pathology of this structure.

I wish to congratulate Mr. Constant on the very scientific manner in which he has presented his paper and the ingenious theory advanced as to the method of eruption of the teeth. I cannot fully agree with him, however, in this matter, since it will not to my mind account for all the phenomena connected with tooth eruption and elongation. His theory of setting up irritation by blood-pressure about the crown is good, but from this point on there are other conditions which must be taken into consideration.

The pathology in erupting teeth is clear. No matter what the propelling force may be, there is an irritation set up around the crown of the tooth in the alveolar process. This irritation produces inflammation. Halisteresis and Volkmann's perforating canal absorption are set up, and room is made in the bone for the advancing crown. The same inflammation, although not quite so severe, sets the bone-cells at work at the border around the root, thus forcing the tooth forward. It is barely possible that these bone deposits are what Pierce intended to illustrate by pounding upon the barrel to remove the bung. If Mr. Constant is correct in his theory, so far as the starting of the crown is concerned, I may agree, but certainly it would not account for the eruption of the tooth after the roots had begun to form. The blood space is so small and the resistance so great that it would appeal to me to be due rather to the bone-cells filling around the roots than to blood-pressure. What is true of the eruption of teeth also holds good in teeth that have lost their antagonism, years after the roots are completely formed.

In regard to root absorption, I do not understand the difference between pathologic and physiologic absorption of roots or bone tissue. Absorption is absorption, regardless of the irritation and inflammation. The only difference I can see is one of degree and form. I have given many years of study to the pathology of the alveolar process, and I think when once understood it is very simple. We must understand, first, its normal condition, which is the key to the whole subject.

The death of the pulp *per se* does not make the root incapable of absorption as suggested by the author, nor does the crown impinging upon the dead root always cause the deflection.

There are three forms of bone absorption,—osteoclast or lacunæ, Halisteresis, and Volkmann's perforating canal absorption. All are brought about by irritation and inflammation. Root absorption is always due to osteoclast or lacunæ absorption. It makes no difference whether the pulp of the tooth is alive or dead, this absorption is the same. If the pulp is dead, the absorption is much greater than when alive, as witnessed in implanted teeth. When the pulp of a temporary tooth is destroyed, or dies, irritation is set up at the end of the root, inflammation of the peridental membrane and alveolar process takes place, and the surrounding bone and trabeculæ or fibrous tissue which holds the lime-salts together is destroyed. Since it is necessary for the trabeculæ or fibrous portion of the alveolar process to be present in order to hold the osteoclasts, it being destroyed, the osteoclasts cannot absorb the roots of the temporary teeth. The remark of Sudduth that the absorbed and the absorber must be in close connection is very apt in this case. The alveolar process having been destroyed, the root of the temporary tooth is only held in position at the cervical margin by the gum and alveolar process. The deflection of the root, therefore, may be simply the result of mastication and not always caused by the crown of the permanent tooth.

We are obligated to Mr. Constant not only for his paper but for his presence at this meeting, and while we are not so many in number, yet I can assure him that the theories advanced in his paper will ever after be associated with his name in relation to this subject in this country. The purely scientific men in this country or abroad are few, but there is this satisfaction to him, as well as to us, that we are trying to advance our specialty along given lines regardless of nationality.

*Dr. Fletcher.*—I would like to ask Mr. Constant why the tooth is not pushed forward after the root is completed?

*Mr. Constant* (replying to Dr. Fletcher).—Dr. Fletcher and Dr. Talbot apparently are at a great difference on the same point. Dr. Talbot says that he quite agrees that he believes the tooth could commence growing, but does not believe that after formed there would be force enough to keep it coming up. That is what I pointed out. In the diagram on the right side of the blackboard there is a broad base of vascular tissue upon which the pulp rests; consequently the lifting force is very great. It is less there (indicating); it is less here (indicating); it is less here (indicating). Still there is a little extrusive force in the peridental membrane. As we find when we get inflammation of the pericementum, you get the tooth raised in the socket. The reason it is not entirely extruded is that while the pulp is alive the alveolus expands it to a certain extent, and by the time the alveolus has gone a little beyond the normal the fibrous tissue in the pericementum has developed to such an extent that it forms a ligament of the tooth, and the development of the fibrous portion of peridental membrane ties the tooth in its socket. I did not explain that in the paper, because I believed it would be obvious without stating. There is an extrusive tendency given by the vascularity of the pericementum.

The Chair ruled that the further discussion of Mr. Constant's paper be postponed until the following morning.

Adjourned to Thursday, May 7, at 10 A.M.

### THIRD SESSION.

The meeting was called to order at 10.15 A.M. by the Chairman, Dr. M. L. Rhein.

The discussion of the paper of Mr. Constant, of England, was continued.

*Dr. M. H. Fletcher,* Cincinnati.—If I understand the condition that Mr. Constant describes, it seems to me that the subject for discussion might be divided into the physical properties and the chemotactic, or, if I may be allowed to use the word, psychical properties, the opposite of the physical. His hydrodynamic theory of the pressure from below by the pumping of the blood, it seems to me, is the most satisfactory explanation we are to have, if we must have a physical explanation for this process of the eruption of the teeth. It is not certain to me that we need such a theory. At the same time, I want to compliment the essayist on the unique features which he presents and to say that they appeal to me and to my method of reasoning better than any theory that I have heard. If we accept his hydrodynamic theory of the pumping of the blood into the cavity or reservoir of soft tissues below the hard, it accounts, of course, to quite a degree for the movement of the tooth out of the alveolar process, whether it be above or below. Nevertheless, when a tooth is completed, we have in a sense a more perfect hydraulic press than we would in the other way, aside from the fact that we have a little background. In other words, in the hydraulic press you have a small opening into a chamber. The pressure of blood comes into this, pushing the plunger forward. In this condition the tooth itself could be considered the plunger, and the pressure of the blood-vessels coming in at the apices of the roots would undoubtedly force the tooth out of the socket. If I understand the philosophy of hydrostatics, we have in the body a great many mechanical processes whereby motion is made or extrusion accomplished. The peristaltic motion of the intestines is an example of the extrusion of bodies. At the same time, I do not understand that we have such a condition in the eruption of the tooth. In applying this hydrodynamic force we can account for many of the features from a physical stand-point. I am inclined to look upon this as a process or one of the forces of a body which comes under that of differentiation in the field of electricity and physics, and the law of differentiation so far as organic material is concerned. It is the law with electricians to see that a conductor of electricity moving at the rate of one inch per second through a magnetic field will produce a variety of pressure. We do not see electricity; we only know by its results what we have. We know we have our electric light and power. The eruption of the tooth, to my mind, could be compared with the expulsion of the ovary from the ovum. We have a like movement there. I do not see that we could apply hydrodynamics to that. When this ovum is about to be expelled we have the fimbriæ enclosing that ovary. We can easily understand how, when it is discharged into the Fallopian tube, it may be forced along by the ciliated epithelium. But why do we have the rising to the surface of this ovum? What is it that causes the fimbriæ of the Fallopian tube to grasp the ovary? We have here powers in physiology which to my mind explain more fully the mechanism or the processes of the eruption of the tooth than any mechanical process. In the differentiation of cells we are in the field of the unknown. We accept the facts, and that is as far as we can go. Why is it that when the ovum is discharged there is a force, which I believe might be called chemotactic (we have such a term in chemistry), which moves the male element to the female element? Why is it we have a differentiation of cells or a segmentation of the ovum? What is it that makes this proliferate into three layers of cells, epiblasts, mesoblasts, and hypoblasts, from which all life is developed? Here are forces that seem to me we deal with in the eruption of the tooth. Why, in the bifurcation of the root of the tooth, is it that we have the molar developing to make two or three roots, or why, in the incisor tooth, do we have only a single root? This is a differentiation of structure or of the development of tissue which I do not believe we can account for, but which we must accept just as we accept the theory of the production of electricity by dynamos. To me this chemotactic process or this attraction which one organic element has for another, or this law whereby we have the development of the various tissues from the one cell, is the only one by which we can really account for this process. This mechanical theory is certainly the best I have heard; at the same time, it does not to me account for the eruption of the teeth and their pushing forward. We can readily understand how a tooth may be diverted from its normal tract and still develop. That could be accounted for mechanically, but to account for the eruption of the tooth mechanically seems to me is not in line with the best and most scientific researches in biology. In the study of physiology and the differentiation and development of cells we are obliged to accept these laws. We know that they do certain things, but why they do I do not think we can understand. Again, to cite other plans of movement in the study of cell life, we have the kinetic process. Why do we have the repulsion of one part of the nucleus from the other? There is a kinetic force here which we do not understand. We have simply in the beginning a differentiation of these chromatic bodies. They finally divide. I should be pleased if the theory of Mr. Constant could be accepted as final, but to me the theory of the mechanical eruption of the teeth is not the most scientific way of working it out, although I believe it is probably one step towards the solution. If we can determine that this is really the process by which the teeth are pushed out or that it is a part of the process we are nearer the solution not only of this problem, but of many of the mechanical movements about the body.

*Dr. Hans Pilcher,* Vienna.—I believe with Dr. Fletcher that we do not know all about the evolution of the teeth. We do not know the cause that starts the whole thing. We do not know the cause that starts the first labor-pains, the first movement towards the birth of the child. I believe we know now the mechanical forces which bring about the movement in the tooth, and we are very much indebted to Mr. Constant. The theory of Mr. Constant is so extremely feasible to me that I believe in it to its fullest extent. In some way the process cannot be entirely mechanical. If there is not something that induces an increased pressure in the pulp tissue there would be a similar pressure from the other parts, even though they may consist of hard bone. I believe that we have to rely upon influences of the nervous system for all these things, and we must suspect that the influences from the nervous system by the vasomotor nerves, or the trophic nerves possibly, must have their influence in this particular kind of movement. Then, there is another thing I would like to ask about, whether any of the gentlemen have seen in a case of acute inflammation of the peridental membrane that a tooth did not elongate because the roots had not the conical form. I do not believe there is such a case, with the exception of cases in which there is very great divergence, which would make a mechanical impossibility of the tooth coming out. It seems to me that those teeth in almost all cases elongate, so I believe the increase of blood supply in the peridental membrane may be taken as an explanation of the final coming out of the tooth.

I wish to congratulate Mr. Constant very heartily, and to say that I believe we and the profession at large are very much indebted to him for his interesting paper.

*Dr. A. E. Baldwin,* Chicago.—Of all the theories that have been advanced for the eruption of the tooth, I think we are greatly indebted to Mr. Constant for this one. It appeals to me more strongly than any other. I do not think it explains completely the causation of the eruption, but it approaches a full explanation more nearly than any other. I believe there is no law of dynamics or of science whereby we can fully explain these so-called physiologic laws. The explanation made by Dr. Fletcher of the discharge of the ovum is not fully accepted. The theory that he advanced is not accepted by many physiologists to-day, although it seems to be the most feasible explanation extant. I have long since dismissed as unworthy of consideration the theories advanced regarding the eruption of the teeth, and that advanced by Mr. Constant seems to me the nearest approach to a solution.

*Dr. M. L. Rhein,* New York.—Before asking Mr. Constant to close the discussion, I want to say that, so far as your chairman is concerned, it affords me the greatest feeling of pride and satisfaction to have been the chairman of this Section at a time when this paper has been presented to us. I do not know of anything that would have repaid me more for a visit so far from New York than to listen to Mr. Constant's paper. I felt yesterday at the close of the session that I wanted to give the paper some hours of consideration before expressing my views upon it, and for that reason suggested that the discussion be postponed until this morning. At the meeting yesterday afternoon I felt very much as Dr. Talbot and some of the other gentlemen expressed themselves, —able to recognize the thorough and logical sequences of the author's remarks, and yet with a strong feeling of doubt whether there was not some flaw in the fabric which he had so ingeniously woven and presented to us. It was after leaving the meeting and as I passed up one of the streets, seeing the working of a pile-driver, that I was struck with the forcible comparison between that mechanical force and the one presented by Mr. Constant. I have given the subject a great deal of thought during the night, and feel this morning that I am unequivocally a convert to all the ideas presented in the paper; and, until I see some demonstration contrary to the facts that I know myself to be true, and to what Mr. Constant has told us, I feel like giving thorough allegiance to his views. All through last evening little incidents in practice, especially in pathological conditions, came to my memory endorsing this view.

Our friend from Vienna, Dr. Pilcher, brought up a very interesting question bearing upon this forcing up of the tooth and whether it was more common in conical roots than in teeth with roots turned and twisted. This pathological element, which undoubtedly has a strong bearing on the theory presented by the author in its applicability, also struck me as being a thorough confirmation of Mr. Constant's views. To make matters clear, in comparative anatomy, we all realize how the conical tooth of the rodent will ultimately cause the death of the animal if the occluding tooth is lost, showing the continuation of this force at work, which will ultimately produce such a condition. Of course, the tooth does not have the periosteal ligamentous attachment that we find in the human tooth. In the same way in clinical work on the anterior teeth, especially the superior incisor, one of the most distressing forms of peridental disease is the sudden commencement of elongation of one of the incisor teeth, carrying with it the additional formation of the pericementum of the root itself.

There are two points about this pathological condition that appear to me to strongly confirm the ideas presented to us. The first is the fact that the shape of the roots of these teeth is very conical, and there is nothing in their shape that in itself would prevent such a forcing out of the tooth. The view presented by Dr. Pilcher, and in conjunction with this a number of radiographs that I have taken of such teeth, have shown little nodular deposits upon the pericemental tissues at different points of the root surface. I have a number of these radiographs in my collection. The connection between these nodular deposits and the forcing out of the tooth has never been clear to me until I listened to Mr. Constant's paper. Now it seems almost conclusive to me that this has interfered with the retentive force of the ligamentous tissue that holds the roots in the position they should occupy when they have been properly erupted. The second reason is that I have failed to see one case of this kind in which this process of elongation has not stopped as soon as the pulps of these teeth have been removed; and in a number of these cases I have found, in sealing the ends of the roots, orifices that were larger than those often found in an ordinary tooth. These are some of the instances that keep coming back to me in thinking of the practicable adaptability of these views, and the more I have thought of them the more have I been impressed that Mr. Constant's theory is the most scientific interpretation of the physiological action of eruption of any heretofore presented.

*Mr. Constant* (closing).—First, I must thank you for your very kind remarks and the kind manner in which you have received the theory that I have placed before you. Let me first speak upon the point which Dr. Walker raised. When I received your kind invitation to attend this congress and to read a paper, it was my endeavor to bring to you something original. I had thought it extremely unlikely that a paper published some years ago in a comparatively obscure meeting should have reached these shores, and therefore I thought probably none of you had heard the theory before. It was somewhat of a surprise to me when I came to Philadelphia to see in the papers published in the Dental Section of the University my own name in connection with the theory of the eruption of the tooth. I was therefore somewhat pleased in one way, but dismayed in another, as I feared I should be presenting something with which all were familiar. The tone of the discussion, therefore, has relieved my mind, and I find that the theory to many of you, at any rate, is unfamiliar.

It would be impossible in the short time at my disposal to enter fully into every fact with regard to the eruption of the tooth. All that I desired to point out was that the very simplest fact in the whole problem had been ignored. It was not my intention to enumerate the things we did not know. I think we should require rather a long session for that, and I think therefore that, when we say that we do not know why the ovum comes to the surface of the ovary, certainly there must be something other than the mere mechanical force in connection with the eruption of the teeth. I think there are reasons we all fully recognize, but I do not think they are reasons for putting forward the purely mechanical side and ignoring the other. That brings me to the point raised by Dr. Talbot. Dr. Talbot stated that he did not think there was any difference in the removal of roots of temporary teeth by resorption and by absorption. I think on that point I must join issue. In the first place, the duration of the process is markedly different and the macroscopic aspect of the roots is widely different.

*Dr. Talbot.*—I said in roots that had dead pulps and implanted teeth.

*Mr. Constant.*—I must have misunderstood the point.

Dr. Talbot reads the part of his paper in question.

*Mr. Constant.*—I inferred from that that Dr. Talbot was of the opinion that, whether a temporary tooth was alive or dead, exactly the same process of absorption occurred, but I see that it does not admit of that construction. My contention is that it is rather more than a question of degree. The appearances, so far as I have seen them, seem extremely different in the two instances. In the one case you get very clear, sharply cut nodules of the edge of the root. In the case of the root of a temporary tooth in which the pulp is being destroyed (I am speaking now entirely of the macroscopic appearance) the surface of the dead root is covered with thick membrane and the edges of the absorbed part are smooth. That is what I term the result of pathological process of absorption. The physiological process of resorption which takes place when the pulp is alive differs very markedly, that difference being the sharp margins and the absence of one thickened membrane which has the greasy feel in the other case.

It is a pleasure in a way to find myself in disagreement with Dr. Talbot, because there is so much upon which we are in accord that it is refreshing to find a point or two upon which we can disagree.

Dr. Talbot speaks of the process of the removal of subjacent tissue from the advancing crown as a process of inflammation. I do not like the term inflammation, used in that connection. I do not want to enter into the point of exactly what takes place during the removal of subjacent tissue, as I wish the simpler point to be cleared up first. Until we are in agreement as to the mechanical force which brings the tooth to the surface we can make no progress. Until we can determine the process by which the tooth comes up it is idle to discuss more complex questions. The point I wish to bring before you and which I have succeeded in making clear, is that mechanical part of the process which is the simplest, and it is the part that has been ignored. That there is a mechanical force there can be no doubt in the mind of any dentist who has seen a tooth pressing upon the gum before eruption. It has been urged that the tooth came up simply because the subjacent tissues are removed; but that idea would be dismissed after close observation.

In regard to a corroborative point, I ventured in the paper to touch upon pathological conditions, and I can assure you that since I have worked out this theory, now some years ago, I have met with no pathological condition that has caused the slightest doubt in my mind as to the applicability of the theory. The point that the president mentioned, and which would strongly confirm the view, is that in the case of the sixth-year molar in which the pulp was prematurely destroyed one finds afterwards that that tooth does not come up properly into line as teeth in which the pulps have not been destroyed, and when it does it is not accompanied by any corresponding growth of the alveolaris; that is to say, there is slight extrusion, but not sufficient extrusion to bring it into occlusion with the opposing tooth.

*Dr. Rhein.*—The Chairman, like the old woman getting in the last word, wants to add one point to Mr. Constant's view of corroboration of the removal of the tissue not being sufficient to bring up the tooth. It is the occurrence that we have all seen so often, the premature lancing of deciduous teeth and the reformation of scar tissue even though the lancing is done in the most thorough manner. I simply mention this as a corroborative point and one which is conclusively clear.

(To be continued.)

